# Histones H3 and H4 require their relevant amino-tails for efficient nuclear import and replication-coupled chromatin assembly *in vivo*

**DOI:** 10.1038/s41598-017-03218-6

**Published:** 2017-06-08

**Authors:** Aïda Ejlassi, Vanessa Menil-Philippot, Angélique Galvani, Christophe Thiriet

**Affiliations:** UFIP UMR-CNRS 6286, Epigénétique: prolifération et différenciation, Faculté des Sciences et des Techniques, 2 rue de la Houssinière, 44322 Nantes, France

## Abstract

Concomitant chromatin assembly and DNA duplication is essential for cell survival and genome integrity, and requires newly synthesized histones. Although the N-terminal domains of newly synthesized H3 and H4 present critical functions, their requirement for replication-coupled chromatin assembly is controversial. Using the unique capability of the spontaneous internalization of exogenous proteins in *Physarum*, we showed that H3 and H4 N-tails present critical functions in nuclear import during the S-phase, but are dispensable for assembly into nucleosomes. However, our data revealed that chromatin assembly in the S-phase of complexes presenting ectopic N-terminal domains occurs by a replication-independent mechanism. We found that replication-dependent chromatin assembly requires an H3/H4 complex with the relevant N-tail domains, suggesting a concomitant recognition of the two histone domains by histone chaperones.

## Introduction

In eukaryotes, genomic DNA is associated with proteins to form chromatin. The basic sub-unit of chromatin is the nucleosome composed of a central tetramer of H3/H4, flanked by two heterodimers of H2A/H2B, and the octamer is wrapped by about two superhelical turns of DNA^1^. The core histones are composed of two distinct domains; the fold domain involved in the histone-histone interaction with the nucleosome and the amino-tail domain that extends outside the nucleosome^2, 3^. The histone tail domains have been shown to be post-translationally modified and these modifications are generally believed to be involved in chromatin activity regulation^4^.

During the S-phase of the cell cycle, the genome replicates and, in conjunction with DNA synthesis, chromatin is assembled^5^. The doubling of the genetic material associated with replication requires parental histone dilution and the synthesis of new histones to compact DNA within the nucleus. Using a pulse labeling strategy for studying newly synthesized histones revealed a conserved di-acetylation on lysines 5 and 12 of histone H4 (corresponding to 4 and 11 in *Tetrahymena*) related to chromatin deposition^6^. This high conservation of the deposition-related di-acetylation of H4 suggested that histone acetyltransferase (HAT) is also highly conserved. In contrast, the newly synthesized H3 acetylation pattern in relation to replication presented a weaker degree of conservation. In the protozoan *Tetrahymena*, the partitioning of chromatin activities between two distinct nuclei, i.e. a macronucleus that transcribes and replicates and a micronucleus that only replicates, enabled HAT activities to be distinguished^7^. Preparation of extracts from micronuclei and cytoplasm revealed that the deposition-related di-acetylation of H4 was catalyzed by a type B histone acetyltransferase, which does not acetylate histone in the chromatin form^8^. This type B HAT was first isolated from yeast cytoplasmic extracts and corresponds to a two-subunit holoenzyme with Hat1p as the catalytic subunit and Hat2p^9^. Consistently with the conservation of the deposition-related di-acetylation of H4, Human HAT1 has been identified and presents a high degree of conservation^10^. Like the yeast enzyme, Human HAT1 is composed of two sub-units, Hat1 and RbA-p46. However, the enzyme was shown to have a nuclear localization in the vicinity of replication forks during the S-phase^10, 11^. Furthermore, the chromatin assembly complex, which is composed of the three subunits of CAF-1 (p150, p60 and RbA-p48) and H3/H4 and which promotes replication-dependent chromatin assembly, exhibited acetylation of lysines 5 and 12 of H4^12^. Despite the conservation of the deposition-related di-acetylation of the H4 complex, the function of these modifications in replication remains to be determined.

The high conservation of the deposition-related di-acetylation of H4 suggested that the H4 amino-terminal tail domain was required for replication. However, unexpectedly, the deletion of the N-tail of H4 in yeast was not lethal^13^. Cell viability was compromised only when both H3 and H4 N-terminal tail domains were deleted^14^. Consistently, microinjection of mRNA during the first division of *Xenopus* embryos revealed that histones lacking the N-terminal tail domain were assembled into chromatin^15^. This lack of a requirement of the amino-tail domains of H3 and H4 was also verified *in vitro* ^16^. Reconstitution of the replication-coupled chromatin assembly system and recombinant H3/H4 showed that the absence of the N-terminal tails of H3 and H4 did not affect the assembly or the interaction of the histone complexes with CAF-1. Nonetheless, these experimental approaches either only partly reconstituted the newly synthesized histone assembly or did not clearly distinguish replication-coupled and replication-independent chromatin assembly^14–16^. However, the incorporation of trace amounts of exogenous histones within naturally synchronized *Physarum* cells proved powerful in overruling the experimental bias^17^. As this approach distinguishes the histone nuclear import and chromatin assembly, it is possible to determine the requirements for each process. Hence, incorporation of truncated histones showed that the H4 N-terminal tail domain was required for efficient nuclear import and that lacking the H3 tail domain prevented chromatin assembly^11^.

In addition to the assembly of nucleosomes behind the replication fork, the doubling of genetic material involves the synthesis of new histones and their transportation into the nucleus. It is believed that histones are actively transported into the nucleus and that amino-terminal tail domains might be involved in this process^18, 19^. Genetic analyses revealed a nuclear localization signal (NLS) within the amino-tail of H2A and H2B^20^. However, incorporation of histone dimers lacking the terminal tail domains in *Physarum* did not lead to a nuclear import defect, suggesting that the histone complex presented features that enabled their transportation into the nucleus^17^. Similarly for H3 and H4, while chimerical proteins composed of GST fused to the amino-terminal tail domains led to nuclear import, analyses of the H3/H4 complex in *Physarum* revealed that lacking the H4 amino-tail domain strongly inhibited the recovery of the complex within the nuclear fraction, suggesting that the presence of an NLS within H3/H4 was not sufficient for their nuclear import^11^. Biochemical analyses of the factors involved in the nuclear import of newly synthesized histones exhibited a high degree of conservation for this pathway between eukaryotes^21^.

To gain insights into the mechanism of the histone amino-terminal tail requirement in nuclear import and replication-coupled chromatin assembly, we created chimerical histones by exchanging the amino-terminal tails and the carboxy-terminal core of H3 and H4; we then incorporated the histones into *Physarum* cells in the S-phase. Our analyses showed a critical role of the amino-terminal tail of H3 and H4 in nuclear import. Although the deposition of the exogenous histones in the S-phase of the cell cycle seemed unaffected by the amino-terminal tail position within the H3/H4 histone complex, our data demonstrated that in replication-coupled chromatin assembly, the histone tail domain and the carboxy-terminal domain are not exchangeable.

## Results

### Nuclear import of exogenous histones requires preformed complexes

**Figure 1:**
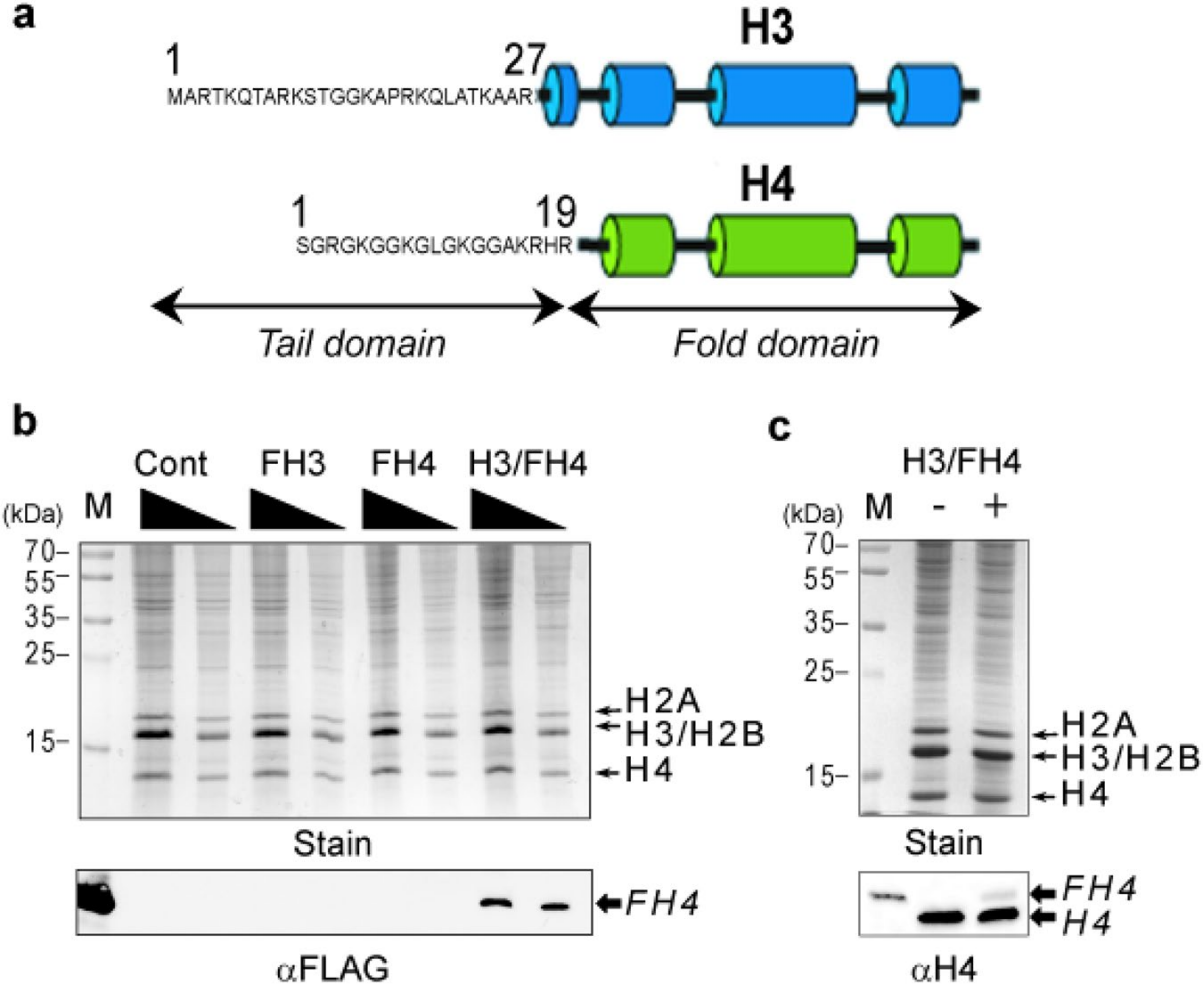
Folded histone complex of H3 and H4 is required for nuclear import in the S-phase. (**a**) Diagram of histones H3 and H4 illustrating the two histone domains; the N-terminal tail domain and the fold domain. (**b**) Nuclear import of exogenous histones requires the correct folding of histones. Trace amounts of purified FLAG-tagged histone H3 (FH3), FLAG-tagged H4 (FH4) and folded complex H3/FH4 (H3/FH4) were incorporated into *Physarum* macroplasmodium fragments in the early S-phase, using an untreated cell fragment as the control (Cont). The cell fragments were then harvested, and nuclei were isolated and analyzed by SDS-PAGE (Stain) and Western blotting with anti-FLAG antibody. Lane M corresponds to the molecular weight marker in (Stain) and the purified H3/FH4 complex revealed by anti-FLAG antibody in (αFLAG). (**c**) Trace amount of exogenous H3/FH4 complex is incorporated into nuclei. Nuclei from cell fragments untreated (−) and treated with H3/FH4 (+) were isolated, analyzed by SDS-PAGE (Stain) and Western blotting revealed with anti-H4 antibodies (αH4). The determination of the amount of exogenous histone relative to endogenous was determined by Western blotting with anti-H4 antibodies. Lane M corresponds to the molecular weight marker in (Stain) and the purified H3/FH4 complex revealed by anti-H4 antibodies in (αH4).

First, in order to determine that exogenous histones were not competing with endogenous histones to form a hybrid complex, histones H3 and H4 containing a FLAG-tag epitope were individually prepared as well as a complex of H3 and FLAG-tagged H4, and then trace amounts of exogenous histones were incorporated into syncytial plasmodium fragments of *Physarum* at S-phase onset. Following 1 h of incorporation in the S-phase, fragments were harvested and nuclei were isolated by Percoll gradient. SDS-PAGE and Western blotting analyses of nuclei were then carried out to estimate the loading and to detect exogenous histones (Fig. 1b). The results revealed that exogenous histones were found in the nuclear fraction only when trace amounts of an exogenous preformed complex of H3/FH4 was spread onto the upper cellular surface of *Physarum*. This indicated that in our experimental conditions unfolded exogenous histones did not efficiently compete with endogenous histones to form a physiologically relevant complex (Fig. 1b), even though endogenous H3 and H4 are abundantly synthesized at this stage of the cell cycle^22^. In contrast to genetic approaches of transfection of histone genes in mammalian cells, the incorporation of trace amounts of exogenous histones (corresponding to < 1% of endogenous histones) into *Physarum* allows the incorporation at precise moment during the cell cycle. Furthermore, the low amount of exogenous histone is inefficient for competing with endogenous histones in the large cytoplasmic volume of *Physarum* macroplasmodium (~3–4 ml) and, therefore, for forming pre-deposition hybrid complexes composed of one exogenous histone and its endogenous counterpart (Fig. 1c). In addition, as shown earlier, trace amounts of exogenous complex were transported into nuclei, and assembled into chromatin with no disturbance of the cell cycle^11, 23^.

### Histone tail domain positioning of H3 and H4 affects nuclear import

**Figure 2:**
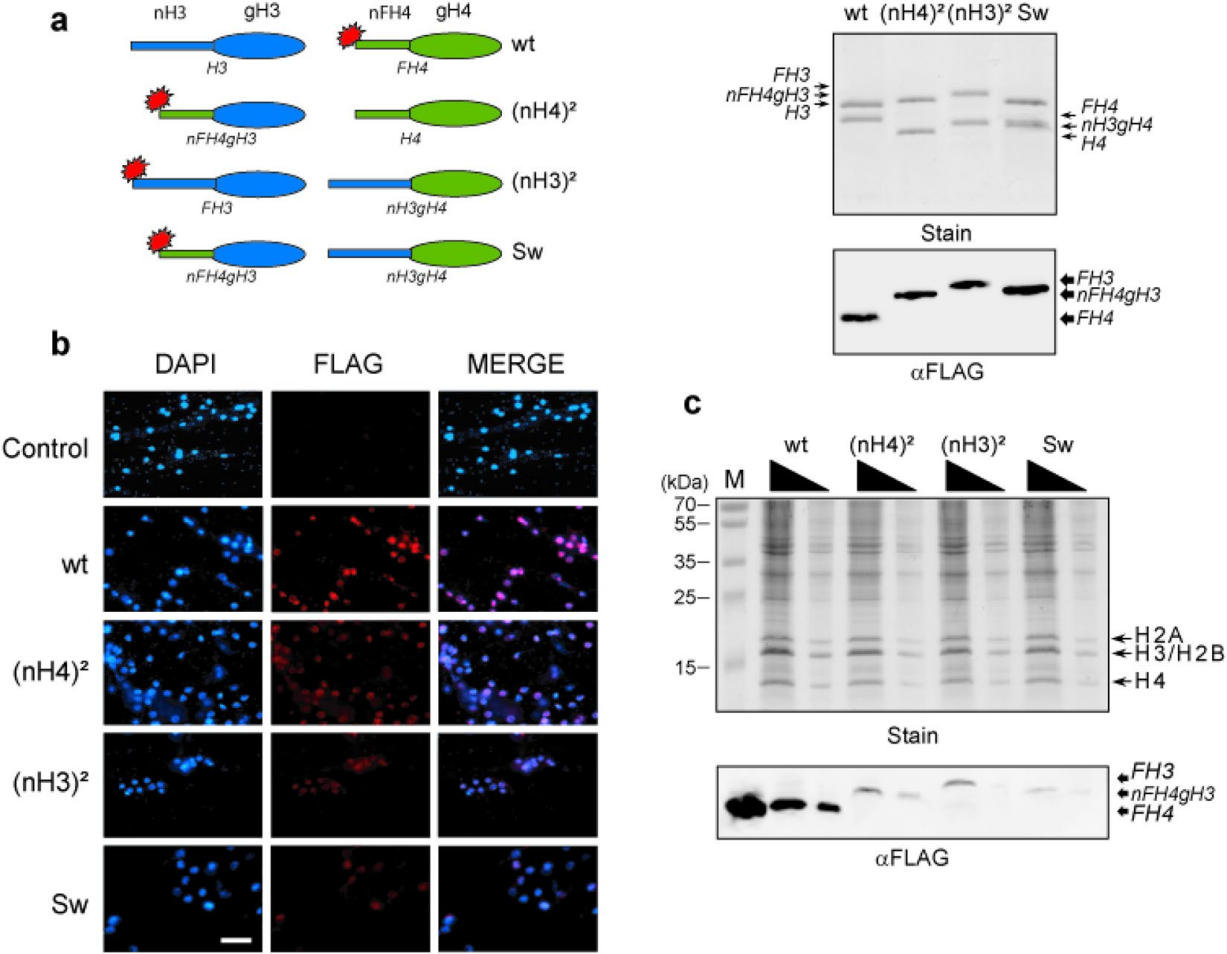
Function of the H3/H4 amino-tail domains in nuclear import in the S-phase. (**a**) Nomenclature and preparation of the exogenous histone complexes. The right panel is a diagram of the different histones used to form the different complexes. The regions in blue correspond to H3 domains, in green to H4 domains, and the red stars represent the FLAG-tag epitope. The complex wt corresponds to H3/FH4, (nH4)² to nH4gH3/FH4 (duplicate of the H4 tail), (nH3)² to FH3/nH3gH4 (duplicate of the H3 tail), and Sw to nFH4gH3/nH3gH4 (swapping of the two tail domains), respectively (the histone sequences are indicated in supplementary information). The right panel shows the SDS-PAGE (Stain) and the Western blot (αFLAG) of the complexes wt, (nH4)², (nH3)² and sw, respectively. (**b**) Microscopic observations of the different H3/H4 complexes. Following incorporation of exogenous complexes, cell fragments were squashed, fixed, and stained with anti-FLAG antibody (FLAG) and counterstained with DAPI (DAPI). The bar corresponds to 20 μm. (**c**) Nuclear import of H3/H4 complexes. *Physarum* fragments were treated during the S-phase with exogenous histone complexes: wt (*H3/FH4*), (nH4)² (*nFH4gH3/H4*), (nH3)² (*FH3/nH3gH4)* and Sw (*nFH4gH3/nH3gH4)*, respectively. Nuclei were then isolated and analyzed by SDS-PAGE (Stain) and Western blotting (αFLAG). Lane M corresponds to the molecular weight marker in (Stain) and the purified H3/FH4 complex revealed by anti-FLAG antibody in (αFLAG).

To determine the function of the histone tail domains of H3 and H4 in nuclear import and chromatin assembly, chimerical histones were prepared in which the amino-tail region and the carboxy-fold domain were swapped. Then histone complexes were folded by combining equal amounts of histones with the carboxy-fold domain of H3 and H4 and presenting a single N-terminal-FLAG epitope (Fig. 2a). The different histone complexes were adjusted to equal amounts and spread onto the upper cellular surface of *Physarum* plasmodium fragments at S-phase onset. To verify the nuclear import of the exogenous complexes, smears of *Physarum* immuno-stained with an anti-FLAG antibody were observed microscopically (Fig. 2b). The different complexes were found to be transported into nuclei with differing efficiencies. Whereas the wild-type complex revealed strong nuclear signals, the ectopic positioning of at least one amino-tail domain significantly reduced the nuclear immuno-staining. It is noteworthy that for microscopic observations of *Physarum* smears, cellular explants are squashed between glass slides and result in cytoplasm dispersion altering cytoplasmic observations. To estimate quantitatively the amount of exogenous complexes in the nuclear fraction, nuclei were isolated and analyzed by SDS-PAGE and Western blotting (Fig. 2c). Consistently with the microscopic observations, the amount of exogenous complexes in the nuclei was affected by the presence of the ectopic tail domain within the H3/H4 complex. In fact, the amount of exogenous complexes presenting one ectopic N-terminal tail (complexes (NH4)² and (NH3)²) was reduced to ~20% relative to the wild-type H3/H4 complex. This reduction was even more pronounced when both H3 and H4 amino-tails were swapped, as less than 10% of exogenous histones were then found in the nuclear fraction. Thus, we concluded that the ectopic positioning of the histone tail domains of H3 and H4 within the complex impedes the accumulation of the folded complex in nuclei.

### Histone tail domain swapping o H3 and H4 do not promote degradation

**Figure 3:**
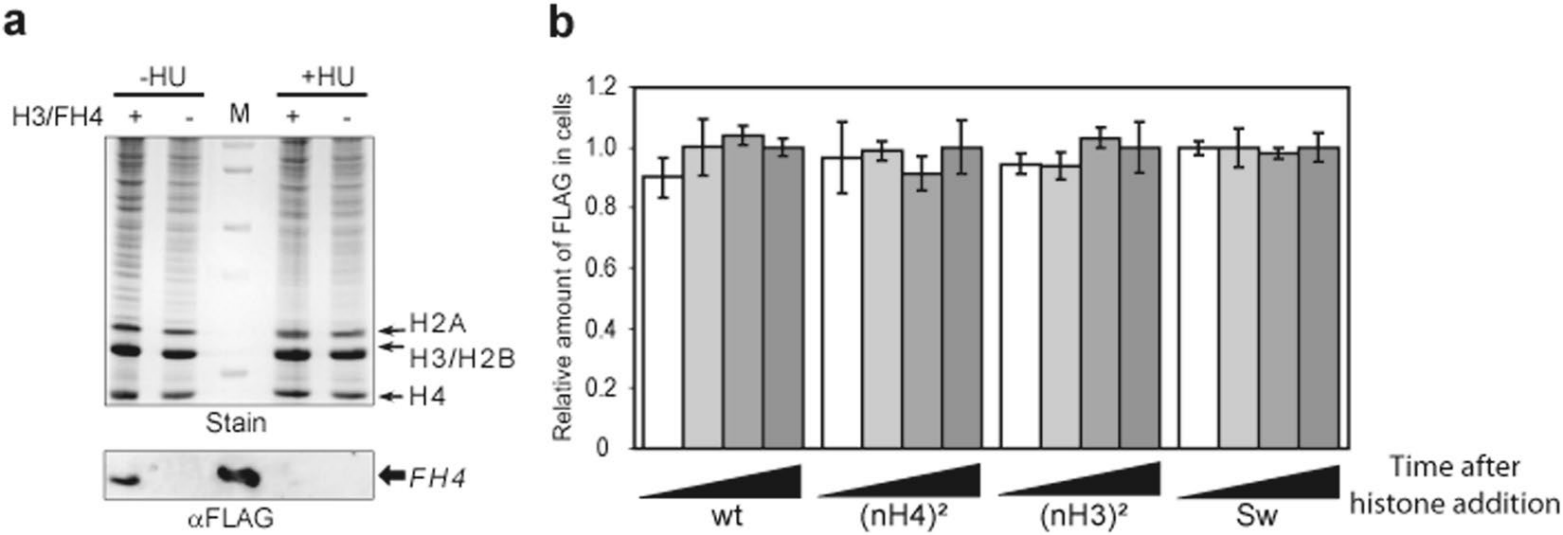
Exogenous histone complexes are stably incorporated into *Physarum* cells. (**a**) Hydroxy-urea treatment inhibits nuclear import. Cell fragments in early S-phase were untreted (−) and treated (+) with exogenous H3/FH4, and untreated (−HU) and treated with hydroxyl-urea (+HU), concomitantly. Nuclei were prepared and analyzed by SDS-PAGE (Stain) and Western blotting (αFLAG). Lane M corresponds to the molecular weight marker in (Stain) and the purified H3/FH4 complex revealed by anti-FLAG antibody in (αFLAG). (**b**) Determination of the stability of exogenous histone complexes in *Physarum*. Cell fragments were treated with HU and exogenous complexes were incorporated for 15 min, 30 min, 45 min and 60 min, respectively. Shown is the quantitative analysis of Flag signal relative to the amount of total soluble proteins determined by dot blotting. The quantification at time point 60 min was arbitrary assigned to 1.0 for each complex. Note that signals of untreated cell fragments with exogenous histones were ~10%. (**c**) Exogenous histone complexes are transported in nuclei with similar rates. The different histone complexes were spread onto *Physarum* surfaces and harvested after 20 min, 40 min and 60 min, respectively. Nuclei were then prepared and analyzed by Western blotting. Shown is the amount of exogenous histone complexes in the nuclear fractions at specific incorporation duration. The value 1 for each complex corresponded to the incorporation after 60 min.

Since our previous experiments revealed that H4/H3 complexes presenting ectopically positioned N-terminal domain did not accumulate into nuclei as efficiently as wild-type complex, we wanted to determine whether the exogenous complexes were rapidly degraded following their incorporation into *Physarum* cells or if the nuclear import of the swapped complexes was inefficient. To verify this, we examined the stability of exogenous complexes in *Physarum*. However, for having comparable situations between the different complexes of H3/H4, we decided to inhibit the H3/H4 nuclear import to prevent differential stable assembly of the complexes in chromatin. We have shown in a previous report that cells treated with HU (Hydroxy-Urea, a replication inhibitor) exhibited a strong inhibition of H3/H4 nuclear import^11^ (Fig. 3a). Thus, incorporations of the different complexes in cell fragments treated with HU were carried out for different durations and the quantities of non-nuclear exogenous histones were determined in total cellular extracts by comparison of the anti-FLAG reactivity to the 60 min time point for each complex. We found that wild type H3/H4 and the swapped complexes were incorporated into *Physarum* cells with similar efficiencies and stably internalized into the cytoplasm over the time course of the experiments event though nuclear import was inhibited (Fig. 3b, Fig. S2). We thus concluded that the different exogenous complexes are neither selectively incorporated nor selectively degraded into *Physarum* cells, but rather present different efficiencies of nuclear import after cellular internalization.

### Positioning of the amino-terminal tail of H3 and H4 affects chromatin distribution

**Figure 4:**
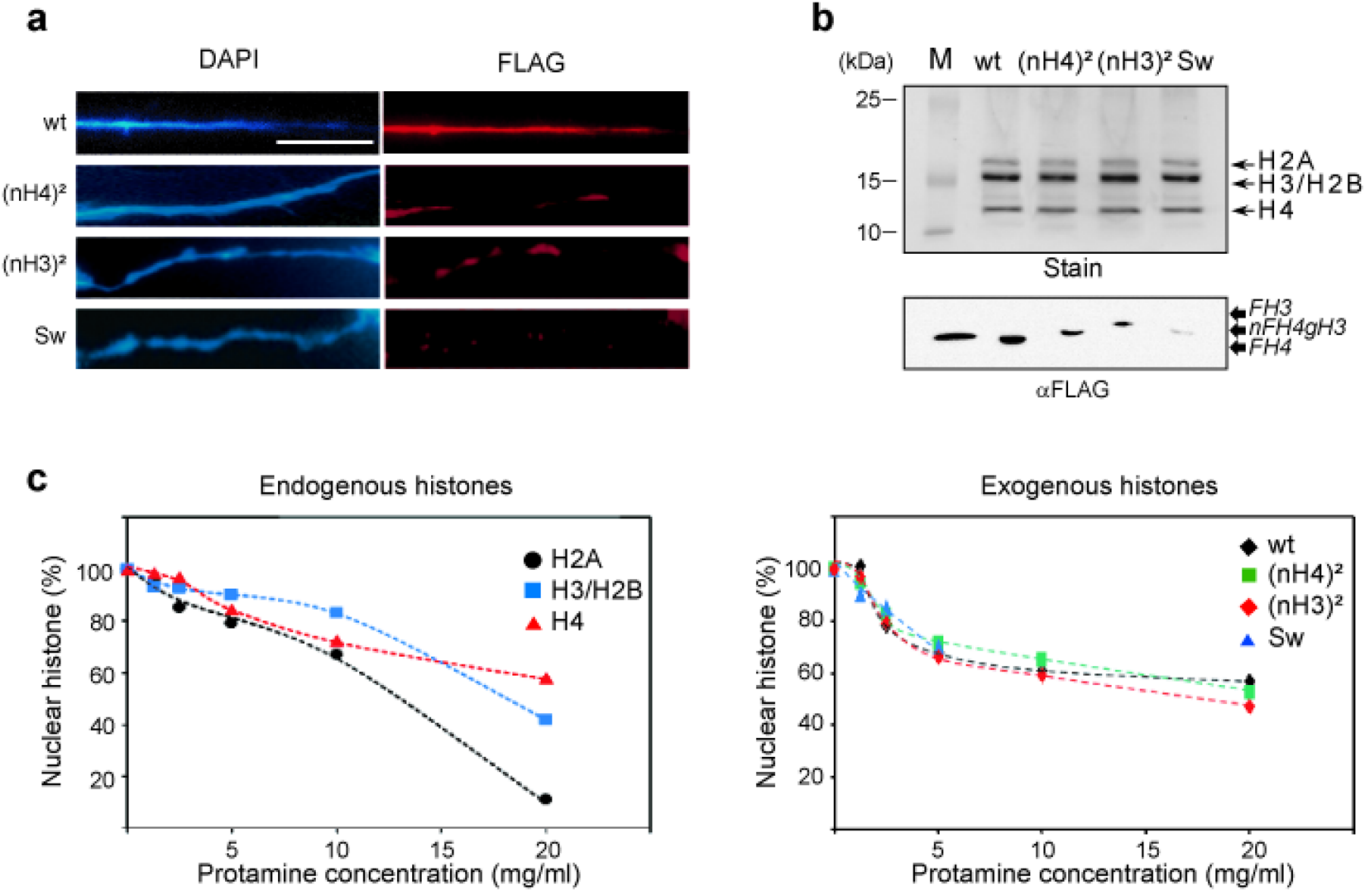
Chromatin assembly in the S-phase occurs regardless of histone tail positioning. (**a**) Microscopic distribution mapping of exogenous H3/H4 complexes. Following exogenous H3/H4 incorporation, nuclei were prepared, chromatin was combed, immuno-stained with anti-FLAG (FLAG) and counterstained with DAPI (DAPI). The chromatin fibers were imaged by fluorescent microscopy. (**b**) Exogenous H3/H4 complexes assembled in nucleosomes. Following incorporation, nucleosomes were isolated from nuclei by MNase digestion and sucrose gradient. Nucleosomal proteins were analyzed by SDS-PAGE (Stain) and Western blotting revealed with anti-FLAG antibody (αFLAG). Lane M corresponds to the molecular weight marker in (Stain) and the purified H3/FH4 complex revealed by anti-FLAG antibody in (αFLAG). (**c**) Histone stability in chromatin. Protamine competition assays were performed on nuclei and analyzed by SDS-PAGE (Endogenous histones) and Western blotting (Exogenous histones), respectively.

Although we have shown that the efficiency of nuclear import was affected by the positioning of N-terminal tail domains of H3 and H4 within the histone complex, we wanted to examine whether the swapping of the N-terminal tails impeded the assembly into chromatin. Chromatin combing analyses were carried out following the incorporation of the different histone complexes (Fig. 4a). The microscopic analyses revealed that exogenous complexes were associated with chromatin. However, chromatin staining revealed different distributions of these complexes. Whereas the wild-type complex presented an even distribution along the chromatin fiber, ectopically positioned amino-tail domains exhibited a punctuated staining of chromatin. These results suggested that wild-type H3/H4 and swapped complexes are associated with chromatin by distinct mechanisms. To verify that exogenous H3/H4 complexes were assembled into *bona fide* nucleosomes, nucleosomes were prepared and the histones were examined by Western blotting (Fig. 4b). Gel staining showed that the amount of nucleosomes was similar in each sample. In contrast, blotting revealed that the amount of exogenous histones in nucleosomes was reduced by the ectopic positioning of the amino-terminal tail domains. However, comparison of the blots of nuclear import and chromatin assembly showed that most exogenous complexes transported into nuclei were assembled into chromatin, suggesting that the deposition of H3/H4 into chromatin is unaffected by the position of the amino-tail domains within the complex (compare Figs 2c and 4b).

*In vivo*, histone modifications and the compaction state of chromatin are believed to affect nucleosome stability and dynamics^24^. We have previously shown by chromatin combing that exogenous histone complexes exhibit distinct patterns of distribution within chromatin. Furthermore, we have shown that the exogenous complexes are assembled into nucleosomes. Thus, in order to verify whether the swapped complexes were assembled into genome regions presenting different nucleosome stability, we carried out a protamine competition assay and examined the release of histones. First, the profiles of protamine competition with endogenous *Physarum* histones were examined by quantifying the histone bands in SDS-PAGE (Fig. 4c, Endogenous histones). Different profiles of release of the endogenous histones were found representing the different histone complexes composing the nucleosomes. Analyses of the H2A band corresponding to the release of the H2A/H2B dimer exhibited a lower stability than the H4 band corresponding to the H3/H4 tetramer, while the H2B/H3 band exhibited an intermediate stability between these two. Then, the profiles of protamine competition in the different exogenous complexes were examined by quantifying Western blot analyses (Fig. 4c, Exogenous histones). The results revealed that the profiles of release of the exogenous complexes from chromatin were closer to that of the endogenous H3/H4 tetramer than other endogenous complexes, except perhaps for the complex carrying the swapped two amino-terminal tail domains of H3 and H4 that could not be accurately quantified above 5 mg/ml of protamine due to the low efficiency of nuclear import and chromatin assembly. Nonetheless, the detailed comparison between the data of endogenous histones and exogenous complexes revealed differences in the curves that might be explained by detection methods used for the quantifications (gel staining for endogenous histones and Western blotting of the FLAG for exogenous complexes). Overall, these experiments suggested that the swapping of the histone tail domains of H3 and H4 did not lead to the assembly of the exogenous complexes within chromatin regions presenting specific nucleosome stability.

### Chromatin assembly in the S-phase occurs by distinct mechanisms

**Figure 5:**
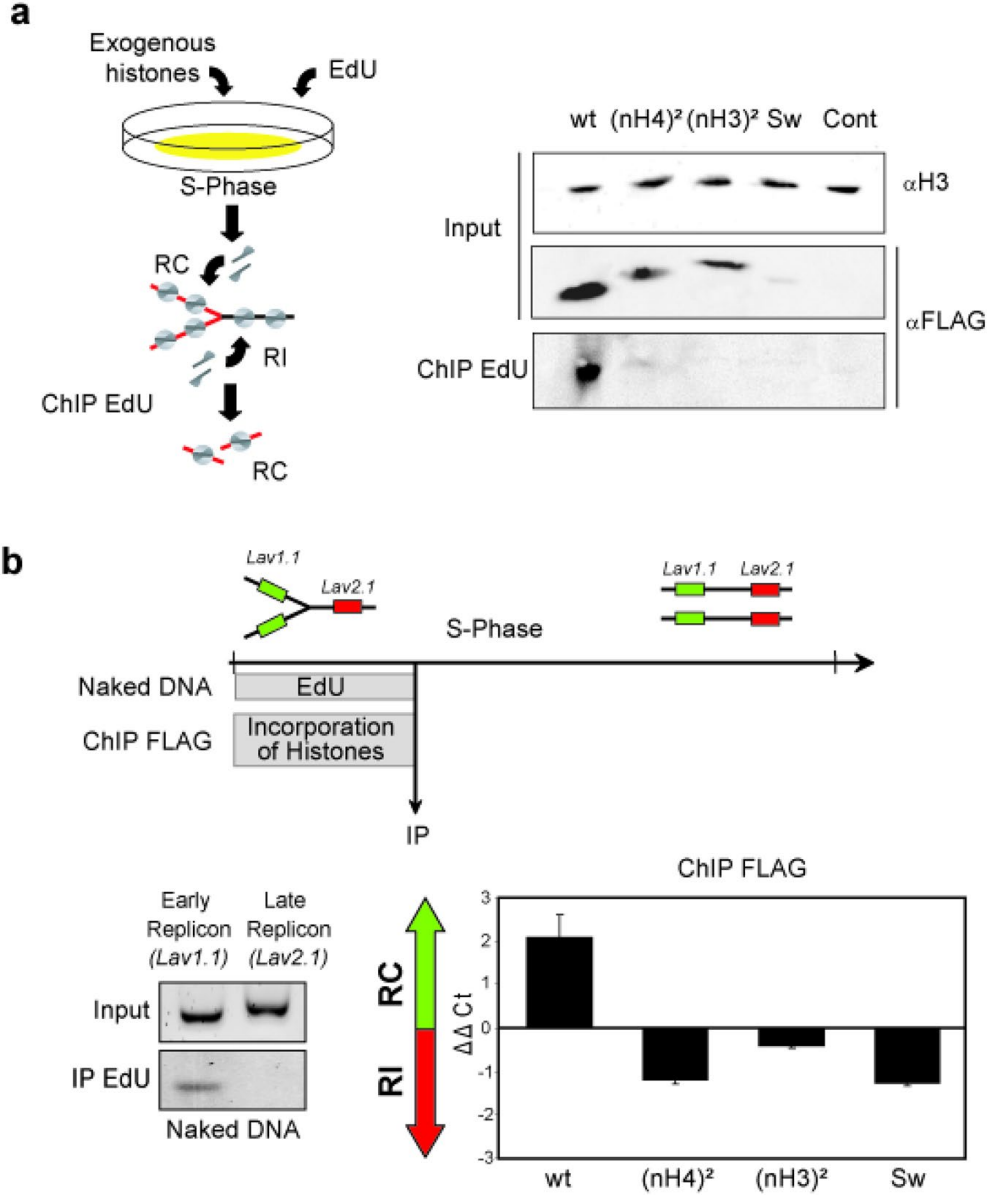
Replication-coupled chromatin assembly requires properly positioned amino-tail domains for transferring H3/H4 from the nuclear import chaperone to the chromatin assembly complex. (**a**) Chromatin assembly in the S-phase occurs by replication-coupled and replication-independent chromatin assembly, which can be discriminated by EdU pulse as depicted in the diagram. Following exogenous H3/H4 complex incorporation, click reactions were performed to couple biotin to EdU and ChIP was carried out with avidin. Crosslinks of immuno-precipitated chromatin were reversed and proteins analyzed by Western blotting. Input fractions were examined with anti-H3 antibody (H3) and anti-FLAG antibody (FLAG), respectively. Immuno-precipitated fractions (ChIP EdU) were examined with anti-FLAG antibody (FLAG). (**b**) Replicon-specific analyses of the H3/H4 tail requirement in replication-coupled chromatin assembly and in replication-independent chromatin assembly. The top diagram represents the experimental strategy for examining replication-coupled and replication-independent chromatin assembly at specific loci. (Left gels) *Physarum* was pulsed for 1 h with EdU and replicated DNA was isolated by IP with avidin. Immuno-precipitated DNA was analyzed by PCR with primers specific to *Lav1.1* (early replicon) and *Lav2.1* (late replicon), respectively. (Right graph) Following exogenous complex incorporation, ChIP analyses were carried out using anti-FLAG antibody and qPCR with primers specific to the early replicon (*Lav1.1*) and the late replicon (*Lav2.1*), respectively. The graph represents the ΔΔCt = ΔCt (ChIP Late replicon normalized) −ΔCt (ChIP Early replicon normalized) for the different exogenous complexes. Value > 0 corresponds to preferential replication-coupled chromatin assembly (RC) and values < 0 corresponds to preferential replication-independent chromatin assembly (RI), respectively.

Our previous experiments showed that the different exogenous complexes of H3/H4 are assembled into chromatin during the S-phase and with similar stability to endogenous histones. However, the chromatin combing experiments showed that the wild-type complex and the swapped complexes presented distinct distributions in chromatin. Thus, we speculated that this difference was related to chromatin activities. As it is known that transcription and repair as well as replication occur during the S-phase, we wanted to determine whether the difference in distribution of the H3/H4 complexes was related to replication-coupled chromatin assembly. To perform these analyses, cell fragments in early S-phase were concomitantly pulsed with EdU (an analogue of thymidine that can be coupled to a moiety by click chemistry) and exogenous histone complexes were incorporated (Fig. 5a). Then, fixed and shared nascent chromatin was precipitated using a biotin moiety coupled to EdU. After cross-link reversion, exogenous histones associated with nascent chromatin were analyzed by Western blotting, using as a control, ChIP products of cell fragments untreated with exogenous histones and EdU. The analyses of the input fractions with anti-FLAG antibody revealed that the amounts of exogenous complexes were consistent with that observed in the nucleosomes (compare Fig. 5a Input/αFLAG and Fig. 4b). Further analyses of exogenous complexes precipitated by ChIP EdU showed that nascent chromatin associated preferentially with wt H3/H4 complex compared to complexes wherein one N-tail domain was swapped. When the two N-tail domains were swapped, this approach did not provide conclusive results as the amount of this exogenous complex was below detection threeshold. These results suggested a role of the position of the N-tail domains within H3/H4 complex in replication-coupled chromatin assembly.

To confirm that replication-coupled chromatin assembly of H3/H4 required the relevant N-tail domains of H3 and H4, we examined the assembly of the exogenous complexes on two specific model replicons presenting precisely defined replication timing^25, 26^ (Fig. 5b, Table S1). To verify that the different replication timing of the loci can be effectively discriminated, cell fragments were pulsed with EdU for 1 h in early S-phase and replicating DNA was coupled with biotin, precipitated by streptavidin-magnetic beads and amplified by PCR with specific primers. The gel analyses of the PCR products showed that within the first hour of the S-phase, only the early replicon (*Lav1.1*) was replicated, consistently with previous reports^25, 26^. Furthermore, the absence of amplification of the late replicon (*Lav2.1*) confirmed that the replication within the entire nucleus population within a single cell is timely regulated as reported^27^. Cell fragments were then treated with exogenous complexes for 1 h, and ChIP analyses were carried out using the anti-FLAG antibody to immuno-precipitate chromatin containing exogenous histones, using as a control cell fragments with no incorporation of exogenous histone. While quantitative PCR analyses of the ChIP of control cells did not allow the detection of specific amplicons for *Lav1.1* and *Lav2.1* (Ct > 40, data not shown), the analyses of cells treated with exogenous H3/H4 complexes revealed that the wild-type complex was enriched in the early replicon relative to the late replicon. In contrast, complexes with swapped amino-terminal domains presented a lower occupancy of the early replicon than the late replicon. These results strongly suggested that replication-coupled chromatin assembly required correctly positioned amino-tail domains and that the assembly of tail-swapped complexes occurred preferentially by a replication-independent mechanism.

## Discussion

In the present work, we determined the effects of the positioning of the amino-terminal tail domain of H3 and H4 on nuclear import and chromatin assembly during the S-phase of *Physarum*. Pioneering experiments using powerful yeast genetics have demonstrated the critical functions of the amino-terminal regions of histones in living cells^14, 28^. Although the growth defects in yeast strains expressing mutated histones might reflect a function in the S-phase, the exact role of these domains in replication has not been elucidated. The natural synchrony of millions of nuclei contained within a single cell of *Physarum* is useful for studying nuclear events at a defined cell cycle stage without artificial synchronization and allows the study of specific processes at precise moments within the 3 h of the S-phase^26, 29^.

In addition to natural synchrony of millions of nuclei, *Physarum* has the unique ability to internalize exogenous proteins spontaneously^29–32^. We have previously used this feature to incorporate trace amounts of exogenous histones, which can be discriminated from endogenous histones and prevent any significant disturbance of the cell cycle progression^23, 24^. This led us to determine the requirement for the histone amino-terminal tail domains in nuclear import and partially in chromatin assembly during the S-phase^11^. In contrast to genetic approaches, the incorporation of trace amounts of exogenous histones in *Physarum* enabled quantitative analyses of nuclear import and chromatin assembly and avoided the deleterious genetic effects. Indeed, genetic depletion or replacement of histone tail domains affected cell viability and did not provide complete information about the tail requirement in nuclear import and chromatin assembly *in vivo* ^14^. The development of GST-histone tail domain fusion proteins in yeast revealed that the histone amino-termini present a nuclear localization signal^19, 20, 33^. However, incorporation of histones into *Physarum* showed that the NLS was not sufficient for nuclear import of histone complexes, suggesting that features within the histone complex are involved in this process^11, 17^. This idea is consolidated by our current data showing that monomers of histones H3 and H4 are not transported efficiently into nuclei while the H3/H4 complex is found in nuclei following incorporation in the S-phase. In addition, our results showed that exogenous histone complexes are not degraded in the time frame of our experiments even if the cellular metabolism did not efficiently utilize the exogenous complexes.

The results of incorporation of exogenous histone monomer in the *Physarum* model system exhibited differences with commonly used cell transfection of histone genes. Indeed, our experiments revealed that the nuclear import of unfolded monomers of H3 and H4 is inefficient compare to folded complex of H3/H4, while the transfection of a single histone gene is sufficient for detecting of the expressed histone in chromatin^34–36^. Most likely, the discrepancy between the biological models is due, at least in part, to different amounts of exogenous histone incorporated into *Physarum* and expressed in transfected cells. In our experiments in *Physarum*, we estimated that the exogenous histones correspond to < 1% of the endogenous ones, which is probably not a sufficient amount for competing endogenous histones efficiently, while this ratio exogenous/endogenous histone is much higher in transfected cells^35, 36^. Moreover, unlike the injection of mRNAs into *Xenopus* eggs that formed hybrid complexes composed of endogenous and exogenous histones and provided mutations to be compensated^15^, the incorporation of the histone complex in *Physarum* prevented competition of exogenous histones with endogenous ones and, therefore, allowed unambiguous determination of the function mutation within the H3/H4 complex in nuclear import.

Our analyses of the role of the amino-tail domains of H3/H4 showed that the correct positioning of the N-tails of H3 and H4 within the complex is required for replication-coupled chromatin assembly. These data contrast with *in vitro* experiments, which demonstrated that lacking the tail domains of H3/H4 did not affect nucleosome formation in replication-coupled nucleosome assembly by CAF1^16^. However, isolation of histone-containing CAF1 (CAC) exhibited the conserved replication-related di-acetylation of H4^12^. In addition, *in vivo* data were more elusive despite the high degree of phylogenic conservation in H3 and H4 and histone chaperones suggesting a similar mechanism in eukaryotes, probably due to the requirement of the successive nuclear import and chromatin assembly of newly synthesized histones in living cells^14, 15^. Our results support distinct partners in these processes, as the different H3/H4 complexes did not exhibit a similar efficiency in nuclear import and replication-coupled chromatin assembly, suggesting that histone complexes are transferred from nuclear import to chromatin assembly complexes. It has been shown that HAT1 is involved in the nuclear import of newly synthesized histones and is in the vicinity of replication forks^11, 21^. Interestingly, recent crystallization and biochemical data showed that in addition to HAT1 binding with the first helix of H4, it presented a high affinity for H4 and H3 amino termini, consistent with our results highlighting the requirement of a properly positioned fold domain with the relevant tail^37^. In contrast, our results showed that folded complexes presenting swapped N-terminal tails preferentially assembled in chromatin in replication-independent manner in S-phase. Even though it is known that replication-independent chromatin assembly occurs outside S-phase^24, 38^, it would be interesting in the future experiments to verify whether the positioning of the N-tail domains of H3.3 containing complex is dispensable outside S-phase.

## Methods

### Culture of Physarum polycephalum

*Physarum*, strain TU291, was cultured as previously described^29^. The cell cycle progress and the synchrony of nuclei within macroplasmodia was monitored by observation of mitosis by phase contrast microscopy^29^. *Physarum* lacks the G1-phase while the S-phase occurs just after mitosis and lasts 3 h.

### Preparation of histones and incorporation into *Physarum*

Genes coding for Xenopus H3 and H4 were modified by PCR and cloned into pET3a, and the proteins were overexpressed in bacteria and purified^39^. Histone complexes were formed by mixing individual histones at stoichiometry and the mixtures were dialyzed in a refolding buffer^40^ (histone sequences are indicated in supplementary information).

For the incorporation of histones into *Physarum* cells, macroplasmodia were cut into fragments of equal size and a solution containing trace amounts of histone proteins (<1%) was deposited on the upper surface^29, 39^. The incorporation was performed at the beginning of the S-phase of the cell cycle and treated plasmodia were kept in growth medium in the dark at 26 °C for 1 h unless indicated in the text. EdU pulses, click conjugation of avidin and precipitation with streptavidin magnetic beads were carried out accordingly^41^.

### Isolation of nuclei and preparation of chromatin

Macroplasmodium segments on filter paper supports were washed in 5 mM EDTA. For total cell analyses, plasmodium fragments were sonicated in 10 mM Tris-HCl, pH 8.0, 1% SDS. The cell lysates were then centrifuged at 25,000 g for 1 min and the supernatants were used for the analyses. The nuclei isolation was performed as following: The cells were then harvested and disrupted by Dounce homogenization in isolation buffer^11, 23^. The nuclei were pelleted by centrifugation at 700 g for 5 min. The nuclear pellet was resuspended in Percoll-containing isolation buffer (same composition as isolation buffer with 25% Percoll) and the suspension was transferred into ultracentrifuge tubes and spun for 40 min at 40,000 g in a Ti90 rotor. The nuclei were then collected in the bottom of the tubes, transferred into 12-ml tubes, washed with isolation buffer, and pelleted by centrifugation. Nucleosomes were prepared by MNase digestion of nuclei as previously described^17^, followed by fractionation of 5%–20% sucrose gradients. ChIP analyses were performed using the standard procedure as described in ref. 24. Biotin conjugation to EdU with click reaction was carried out by mixing chromatin with biotin azide in a Cu/THPTA/Ascorbate buffer for 1 h. Avidin precipitation was performed using streptavidin magnetic beads according to the manufacturer’s instructions (Ademtech).

### Fluorescent microscopy and chromatin fiber combing

Fluorescent microscopic observations of nuclei and chromatin fibers were made using a Nikon Ni-E. For the observations of nuclei, smears of *Physarum* explants fixed with ethanol were revealed with anti-FLAG antibody and the appropriate secondary antibody, and mounted in glycerol/ethanol (1:1). Samples were then illuminated with the appropriate wavelengths to visualize either DAPI-stained DNA or rhodamine-labeled exogenous proteins. Chromatin fiber combing was performed as described in ref. 42. Specifically, Percoll gradient purified nuclei were resuspended in PBS and deposited on siliconized microscopy slides in the presence of 10 mM EDTA. Nuclei were spotted on microscopy slides, incubated for 12 min in lysis buffer and the buffer was removed linearly to extend chromatin fibers at the air/liquid interphase. The fibers were then fixed with 4% formaldehyde in PBS for 10 min and extracted in permeabilization buffer for 10 min. The slides were then treated with antibodies to visualize the exogenous histones.

### Protamine competition assay

Protamine competition assays were performed on Percoll gradient isolated nuclei^43, 44^. Nuclei were quantified in 2 M NaCl/4 M urea by spectrometry at 260 nm. Nuclei corresponding to 1 OD were resuspended in 400 μl PBS and protamine was added; 250 μg, 125 μg, 62.5 μg, 31.25 μg, 15.625 μg, and 7.8125 μg, respectively. The suspensions of nuclei were then kept on ice for 1 h. Nuclei were pelleted by centrifugation at 10,000 g for 2 min and analyzed by SDS-PAGE and Western blotting.

## Electronic supplementary material

Supplementary information
