# Supplementary material for: Histones H3 and H4 require their relevant amino-tails for efficient nuclear import and replication-coupled chromatin assembly in vivo
Source: Sci Rep. 2017 Jun 8;7:3050. doi: 10.1038/s41598-017-03218-6 (PMC5465201; doi:10.1038/s41598-017-03218-6)
Supplement: Supplementary file 1 — Supplementary information [file 41598_2017_3218_MOESM1_ESM.pdf]

Histones H3 and H4 require their relevant amino-tails for efficient nuclear import and replication-coupled chromatin assembly *in vivo*

Aïda Ejlassi†, Vanessa Menil-Philippot†, Angélique Galvani, Christophe Thiriet*

UFIP UMR-CNRS 6286, Epigénétique: prolifération et différenciation, Faculté des Sciences et des Techniques, 2 rue de la Houssinière, 44322 Nantes.

*: Christophe.Thiriet@univ-nantes.fr

†: These authors contributed equally to this work

FH3

MDYKDDDDKPWARTKQTARKSTGGKAPRKQLATKAARKSAPATGGVKKPHRYRP
GTVALREIRRYQKSTELLIRKLPFQRLVREIAQDFKTDLRFQSSAVMALQEASEAYLV
ALFEDTNLAAIHAKRVTIMPKDIQLARRIRGERA

H3

MARTKQTARKSTGGKAPRKQLATKAARKSAPATGGVKKPHRYRPGTVALREIRRYQ
KSTELLIRKLPFQRLVREIAQDFKTDLRFQSSAVMALQEASEAYLVALFEDTNLAAIH
AKRVTIMPKDIQLARRIRGERA

nFH4gH3

MDYKDDDKSGRGKGGKGLGKGGAKRHKSAPATGGVKKPHRYRPGTVALREIRRYQ
KSTELLIRKLPFQRLVREIAQDFKTDLRFQSSAVMALQEASEAYLVALFEDTNLAAIH
AKRVTIMPKDIQLARRIRGERA

H4

MSGRGKGGKGLGKGGAKRHRKVLRDNIQGITKPAIRRLARRGGVKRISGLIYEETRG
VLKVFLENVIRDAVTYTEHAKRKTVTAMDVVYALKRQGRTLYGFGG

FH4

MDYKDDDKSGRGKGGKGLGKGGAKRHRKVLRDNIQGITKPAIRRLARRGGVKRISG
LIYEETRGVLKVFLENVIRDAVTYTEHAKRKTVTAMDVVYALKRQGRTLYGFGG

nH3gH4

MARTKQTARKSTGGKAPRKQLATKAARRKVLRDNIQGITKPAIRRLARRGGVKRISG
LIYEETRGVLKVFLENVIRDAVTYTEHAKRKTVTAMDVVYALKRQGRTLYGFGG

Figure S1: Histone sequences. Shown is the sequence of the histones used for generating the different complexes. Highlighted in red is the FLAG sequence, the amino-acids in blue correspond to H3 and the amino-acids in green correspond to H4.

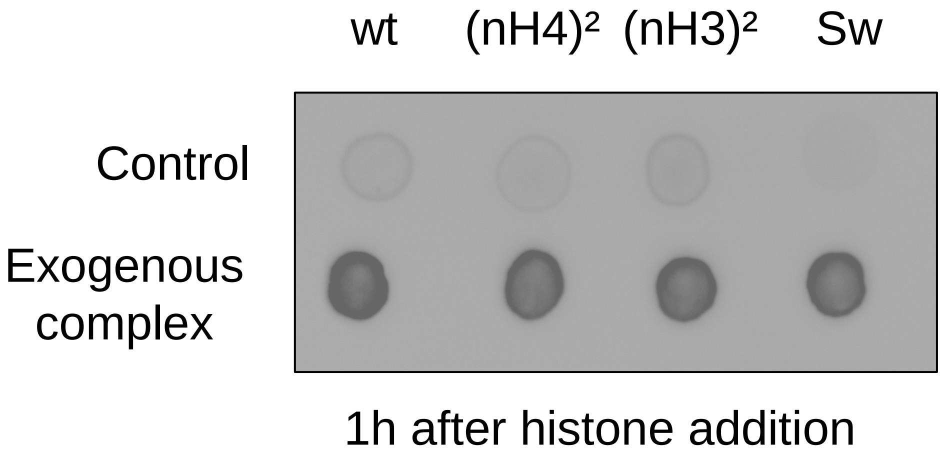

Figure S2: Dot blot analyses of cell lysates. Cell fragments cultured in presence of Hydroxy-Urea were untreated (Control) and treated (Exogenous complex) for 1h at the onset of S-phase with exogenous complexes, wt, $(nH4)^2$, $(nH3)^2$ and Sw, respectively. Cellular extracts were prepared and analyzed by dot blotting revealed with anti-FLAG antibody.

	wt	$(nH4)^2$	$(nH3)^2$	Sw
ΔCt early replicon/input	10.9±0.04	11.06±0.04	11.26±0.05	12.01±0.03
ΔCt late replicon/input	12.98±0.05	9.87±0.01	10.84±0.03	10.75±0.02

Table S1: Delta Ct calculation of ChIP experiments: Duplicates of independent incorporation and duplicates of q-PCR analyses were used for the calculations. No specific amplicons were detected from ChIP of control cells untreated with exogenous (Ct > 40).
